# Synergy of Cobalt and Silver Microparticles Electrodeposited on Glassy Carbon for the Electrocatalysis of the Oxygen Reduction Reaction: An Electrochemical Investigation

**DOI:** 10.3390/molecules200814386

**Published:** 2015-08-07

**Authors:** Claudio Zafferoni, Giacomo Cioncoloni, Maria Luisa Foresti, Luigi Dei, Emiliano Carretti, Francesco Vizza, Alessandro Lavacchi, Massimo Innocenti

**Affiliations:** 1 Department of Chemistry, University of Florence, via della Lastruccia 3, 50019 Sesto Fiorentino (Fi), Italy; E-Mails: marialuisa.foresti@unifi.it (M.L.F.); luigi.dei@unifi.it (L.D.); carretti@csgi.unifi.it (E.C.); 2 WestCHEM, School of Chemistry, University of Glasgow, University Avenue, G12 8QQ Glasgow, UK; E-Mail: g.cioncoloni.1@research.gla.ac.uk; 3 Institute of Chemistry of OrganoMetallic Compounds, ICCOM-CNR, via Madonna del Piano 10, 50019 Sesto Fiorentino (Fi), Italy; E-Mails: francesco.vizza@iccom.cnr.it (F.V.); alessandro.lavacchi@iccom.cnr.it (A.L.)

**Keywords:** ORR, electrodeposition, silver, cobalt

## Abstract

The combination of two different metals, each of them acting on different steps of the oxygen reduction reaction (ORR), yields synergic catalytic effects. In this respect, the electrocatalytic effect of silver is enhanced by the addition of cobalt, which is able to break the O–O bond of molecular oxygen, thus accelerating the first step of the reduction mechanism. At the same time, research is to further reduce the catalyst’s cost, reducing the amount of Ag, which, even though being much less expensive than Pt, is still a noble metal. From this point of view, using a small amount of Ag together with an inexpensive material, such as graphite, represents a good compromise. The aim of this work was to verify if the synergic effects are still operating when very small amounts of cobalt (2–10 μg·cm^−2^) are added to the microparticles of silver electrodeposited on glassy carbon, described in a preceding paper from us. To better stress the different behaviour observed when cobalt and silver are contemporarily present in the deposit, the catalytic properties of cobalt alone were investigated. The analysis was completed by the Levich plots to evaluate the number of electrons involved and by Tafel plots to show the effects on the reaction mechanism.

## 1. Introduction

The catalytic properties of silver were reported long ago [1,2,3,4,5,6]. More recently, silver single crystals were used to stress the surface sensitivity of ORR and the pH effect [7,8]. The most appropriate reaction scheme for the ORR provides two different four-electron pathways [7,8,9,10,11,12,13], the first one directly leading to the formation of H_2_O and the second one occurring with an intermediate two-electron step with the formation of H_2_O_2_, part of which is subsequently reduced to H_2_O in a further two-electron step. It must be stressed that the possible formation of H_2_O_2_ must be minimized, both for the lower energy involved (two electrons in place of four) and for the dangerous aggressive effect on the membrane used in fuel cells. The direct four-electron reaction with a very small (0.5%–2.5%) peroxide formation occurs in the entire potential range in 0.1 M KOH, but only at the highest overpotentials in 0.1 M HClO_4_, suggesting a useful utilization of silver in alkaline media. This result has been very recently supported by the surface Pourbaix diagram of the Ag(111) obtained by density functional theory calculations [14].

To completely remove Pt and to replace it with less expensive materials, bimetallic electrocatalysts have been proposed to exploit a synergic mechanism with one metal able to break the O–O bond of molecular oxygen and the second metal effective in reducing the adsorbed oxygen thus formed [15,16,17,18]. The theoretical assumptions have been supported by the experimental evidence of the catalytic activity of Ag–Co mixtures [15,17,18].

Silver substrates can be electrochemically modified at the nanometric scale through electrodeposition of metals able to exert catalytic synergic effects (Co, Ni, Fe) or chalcogenide nanofilms. The synergic effect of Ag–Co has been verified by electrodepositing Co on Ag(111) surfaces so as to form nanometric islands using a template-assisted procedure [18]. In particular, the maximum synergic effect was found to be exerted by a Co amount equal to 30% of Ag, in good agreement with the theoretical previsions [15,16]. Later, a monolayer of Co, or Fe, or Co–Fe mixtures was deposited through electrodesorption-based atomic layer deposition (SEBALD) [19], and their catalytic effects, even though small, were verified [20].

Currently, the research is directed toward further reducing the catalysts’ cost by reducing the amount of Ag, which, even being much less expensive than Pt, still is a noble metal. From this point of view, using a small amount of Ag, Au or Pd together with an inexpensive material, such as graphite, represents a good compromise [21,22,23,24,25]. Electrocatalysts are deposited by more or less complex chemical procedures or by gas phase methods, and only a few references cite electrodeposition [26]. However, electrodeposition represents a good method to limit the amount of depositing metals through the Faraday laws and, actually, allows the formation of thin and homogeneous Ag deposits [27,28,29,30]. With electrodeposition, the morphology and structure of deposits can be changed by simply varying the experimental parameters, such as electrolyte composition, temperature, solutions, pH and the electric variables (either current or potential), while working at ambient temperature and pressure, thus only requiring inexpensive experimental equipment. On this basis, [31] reports the electrocatalytic properties of Ag microparticles electrodeposited on glassy carbon (GC), which, itself, promotes the two-electron pathway leading to the formation of H_2_O_2_ [6].

An electrochemical quartz crystal microbalance investigation has recently shown the formation of cobalt hydroxide simultaneously with cobalt deposition due to the pH variation near the surface electrode caused by the parallel hydrogen evolution reaction [32]. In the alkaline electrolytes and in the potential range used to study ORR by means of the rotating disk electrodes, the deposits of Co are significantly oxidized to Co(OH)_2_ [33,34]. For its part, cobalt hydroxide, while promoting the two-electron pathway in ORR, also shows an autocatalytic mechanism with an intermediate step, where oxygen is again formed [35,36,37].

In the present paper, we verified the possibility of combining the results of [28] and [38], which is to investigate if the electrocatalytic effect of Ag particles could be further increased by adding a controlled amount of Co.

## 2. Results and Discussion

### 2.1. Electrodeposition of Increasing Amounts of Cobalt on GC and Their Effect on ORR

**Figure 1:**
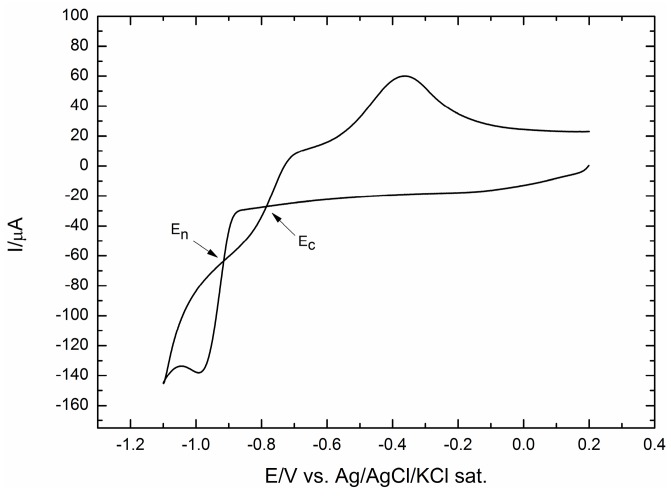
Linear sweep voltammograms for Co deposition from Co(II) (5 × 10^−3^ M) solution in ammonia buffer from 0.2 V–−1.1 V. The scan rate is 50 mV·s^−1^.

Figure 1 shows the cyclic voltammogram of Co on the GC electrode between 0.2 and −1.0 V.

During the forward scan, the cathodic current increases sharply once nucleation starts to occur, since it is related to the increase of the density of the nuclei and the crystal growth [38,39]. During the reverse scan, two crossovers between the cathodic branches’ current are observed. The more negative crossover corresponds to the nucleation overpotential, E_n_, whereas the less negative one is the overcrossing potential, E_c_. In the potential range between E_n_ and E_c_, the cathodic current recorded in the reverse scan is greater than that recorded in the forward one. As reported in [38], this higher cathodic current suggests that the energy required for cobalt deposition on the cobalt film (formed during the forward scan) is lower than that required for cobalt deposition on the bare GC electrode.

**Figure 2:**
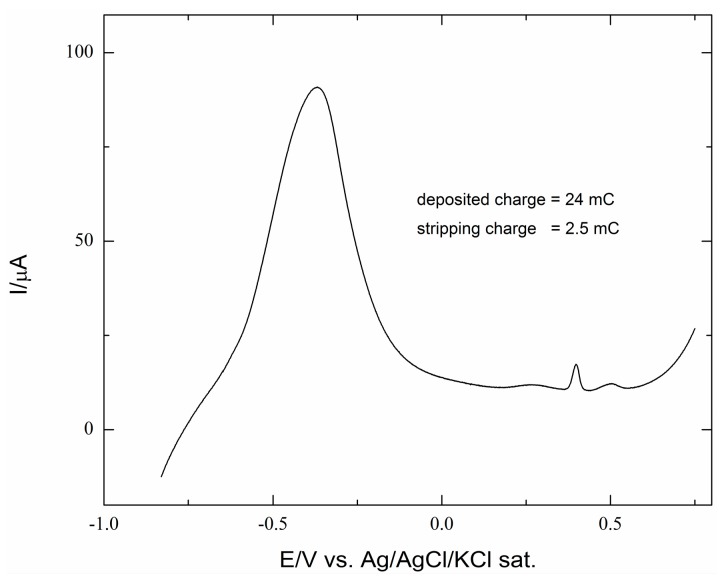
Stripping voltammetry of a Co deposit of 24 mC (equal to 9.3 μg·cm^−2^) as recorded by scanning the potential from −0.8–+0.8 V at a scan rate of 10 mV s^−1^. The small peak at +0.4 V can be associated with the formation of Co(III).

Increasing amounts of Co were deposited at a potential, E = −0.93 V, just a bit negative to E_n_. As for the Ag deposition reported in [31], a purpose-developed program allowed for strictly controlling the extent of deposition. The charge involved in Co(II) reduction was continuously monitored, and as soon as it reached the wanted charge value, the cobalt solution was suddenly replaced by ammonia buffer. However, unlike silver, the electrochemical check of cobalt deposition performed by anodic stripping was distorted by the chemical formation of the solid Co(OH)_2_ on the electrode surface. As an example, Figure 2 shows that only a very small charge, about 2 mC, was involved in the electrochemical reoxidation of a Co deposit corresponding to 24 mC. However, energy dispersive x-ray spectroscopy (EDX) confirms the presence of cobalt in our samples.

**Figure 3:**
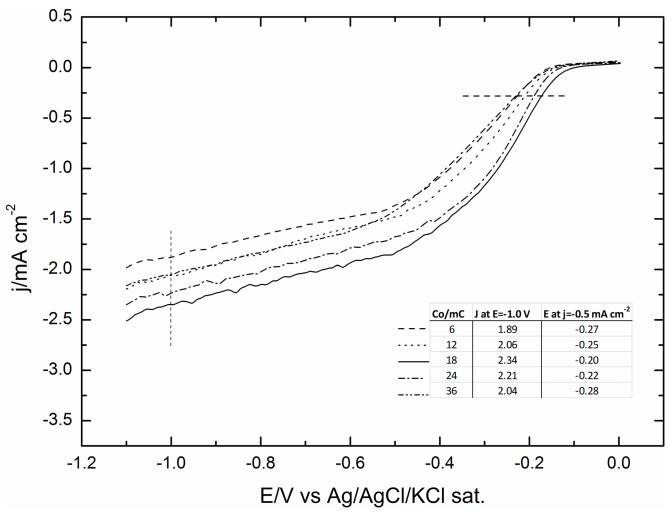
Oxygen reduction reaction (ORR) in KOH 0.1 M on glassy carbon (GC) covered by increasing amounts of Co from 6 mC–36 mC recorded at 500 rpm and with a scan rate of 50 mV·s^−1^.

Incidentally, the current involved in all Co depositions decreases slowly up to a deposited charge of 3.5 mC, and then, it turns to increase constantly, even though slowly, thus suggesting that from that point forward, the deposition occurs on already formed nuclei, rather than on the GC surface. The catalytic effects on ORR of the various deposits of cobalt reported in Table 1 were then analysed. Figure 3 shows the polarization curves (positive sweep direction at 500 rpm) as recorded on a rotating-disc working electrode for the ORR on the various deposits of cobalt reported in Table 1. The figure shows that the onset potential progressively shifts towards less negative potentials up to a deposit equal to 18 mC, and then, it progressively shifts back to the negative potentials for the largest Co deposits.

**Figure 4:**
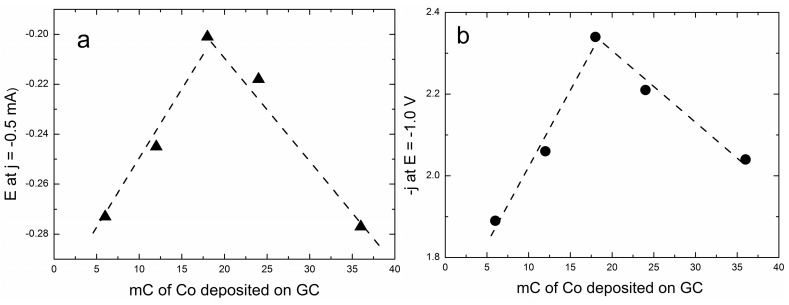
(**a**) Volcano curve as obtained by plotting the potential values at j = −0.5 mA·cm^−2^, and (**b**) the current density values at E = −1.0 V *versus* the charge associated with the Co deposits on GC.

At the same time, the limited cathodic currents follow a similar behaviour. This is well depicted by both the plots of the potentials at which the curves reach the value of 0.5 mA·cm^−2^ and the currents recorded at a potential E = −1.0 V. The result is the volcano curves reported in Figure 4 that are typical of a mechanism where the adsorbed step is the rate-determining step.

**Table 1:** The deposited charges of cobalt and the corresponding amount expressed in μg cm^−2^.

Programmed Charge/mC	Co Loading/μg cm^−2^
6	2.3
12	4.7
18	7.0
24	9.3
36	14.0

Here, the adsorbed species is superoxide ion on Co(OH)_2_, and therefore, the increasing catalytic effect on the left branch of the volcano curve could just be explained by the increased surface of the cobalt deposits. On the other hand, the decrease of the cobalt surface along the right branch could hardly be explained other than with the increased coverage of superoxide occurring in the left branch.

According to [35], the mechanism of ORR on Co(OH)_2_ provides the first pH-independent step:

(1) $$O_{2}\;+e^{-}\to O_{2}^{.-}$$

followed by the disproportionation reaction (2) or the electrochemical step (3):

(2) $$2O_{2}^{.-}+\;H_{2}\text{O}\to {\text{HO}}_{2}^{-}+O_{2}+{\mathrm{OH}}^{-}$$

(3) $$O_{2}^{.-}+\;H_{2}O+e^{-}\to {\mathrm{HO}}_{2}^{-}+{\mathrm{OH}}^{-}$$

In its turn, ${\mathrm{HO}}_{2}^{-}$ formed in Step (3) may still produce O_2_ in Step (4):

(4) $$2{\mathrm{HO}}_{2}^{-}\to 2{\mathrm{OH}}^{-}+O_{2}$$

**Figure 5:**
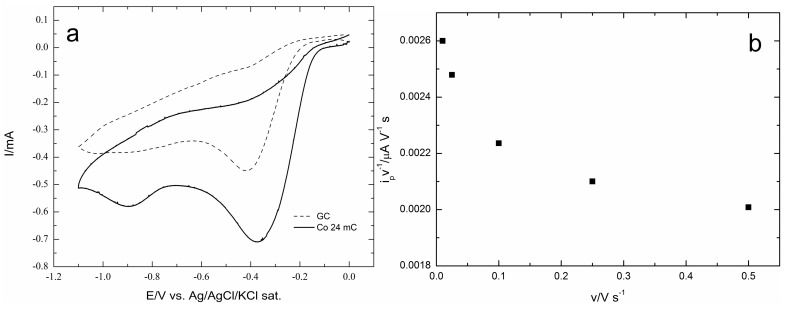
(**a**) Cyclic voltammograms recorded at GC (dashed curve) and at GC/Co(OH)_2_ (solid lines) stationary electrodes; (**b**) i_p_/v ratio as a function of the scan rate v.

Reaction (1) is responsible for the first reduction peak at about −0.4 V that is observed both at the GC and at GC/Co(OH)_2_ stationary electrodes. However, the comparison of the cyclic voltammograms reported in Figure 5a shows that the presence of cobalt hydroxide imparts a high catalytic effect due to re-reduction of O_2_ replenished from Reaction (2) and/or (4). Then, a second ill-defined reduction peak due to the concomitant side two-electron reduction of Step (5) takes place at more negative potentials (at about −0.9 V):

(5) $$O_{2}+\;H_{2}O+2e^{-}\to {\mathrm{HO}}_{2}^{-}+{\mathrm{OH}}^{-}$$

Cyclic voltammograms recorded at different scan rates, v, at the GC/Co(OH)_2_ electrode allow one to verify that i_p_/v decreases with v, thus confirming the autocatalytic mechanism imparted by the presence of cobalt (Figure 5b).

### 2.2. Electrodeposition of Increasing Amounts of Cobalt on GC Covered by Silver Microparticles

To verify the existence of a synergic effect of Co on the silver microparticles of [31], amounts of Co similar to those used in the preceding paragraph were added to the silver deposit of 12 mC (corresponding to 17.1 μg·cm^−2^) that had given the best catalytic effect. In the first series of measurements, we left out the activation treatment on silver microparticles that had conferred enhanced catalytic effects to the silver microparticles. This allowed investigating if the synergic effect of Co on silver, already reported in [18], still worked if silver were in the form of microparticles.

**Figure 6:**
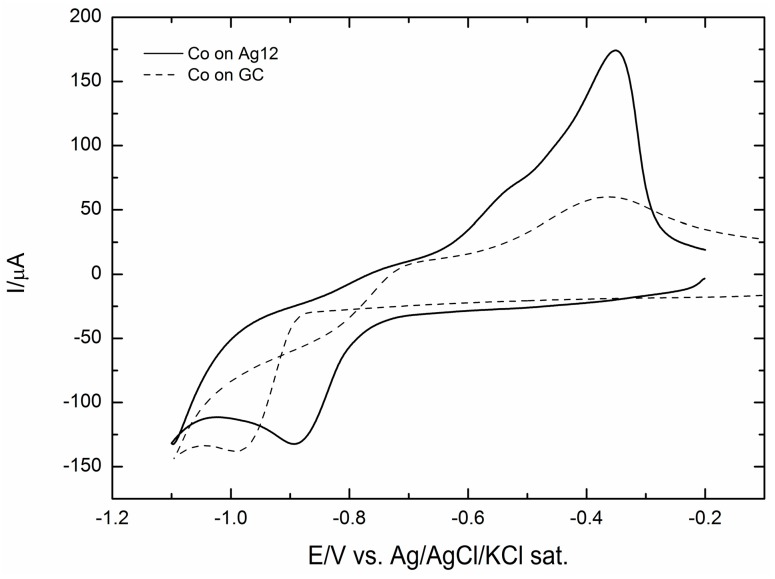
Cyclic voltammograms of Co(II) solution (5 × 10^−3^ M) recorded at the GC electrode (dashed curve) and at the GC/Ag12 mC electrode (solid line).

Figure 6 shows that the electrodeposition of Co on a GC substrate already covered by Ag microparticles is almost different from that on the bare GC electrode. In fact, there are no more intersection points, and the onset of the cathodic peak shifted to less negative potentials. It must be noted that the presence of an overcrossing potential E_n_ is a major parameter in the classification of the characteristics of the nucleation growth mechanism [39]. Therefore, the absence of such an overcrossing potential may tentatively suggest that Co deposition takes place apparently without creating new nuclei.

**Figure 7:**
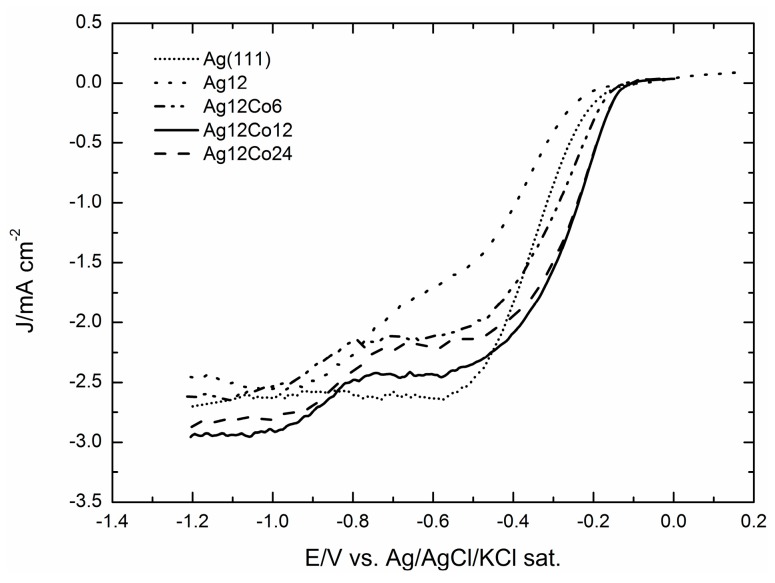
Comparison between the oxygen reduction reaction (ORR) on the Ag(111) electrode and on the GC/Ag12 electrode [31] and the GC electrode covered by an increasing amount of Co recorded at 500 rpm and with a scan rate of 50 mV·s^−1^.

Figure 7 shows the polarization curves (positive sweep direction at 500 rpm) as recorded on a rotating-disc working electrode for the ORR on the above deposits of cobalt together with the curve of the only deposit of 12 mC of Ag.

**Figure 8:**
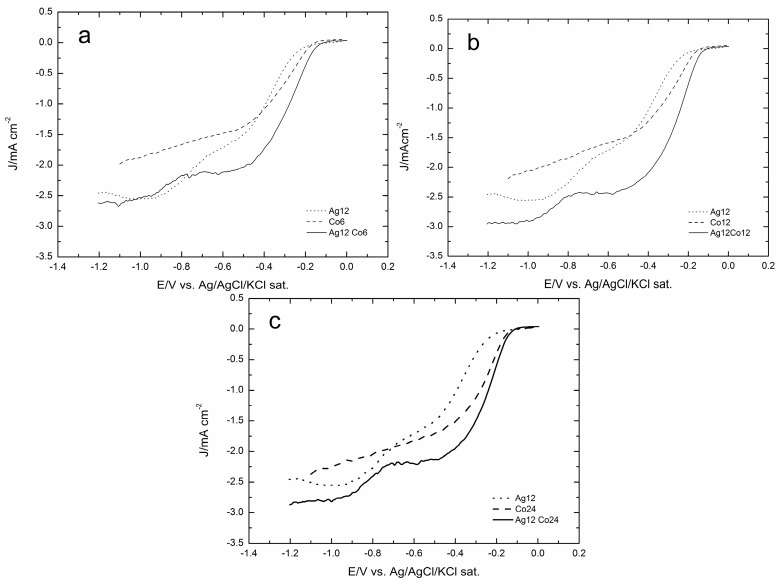
The oxygen reduction reaction (ORR) of the GC/Ag12Co samples and the corresponding GC/Co samples with the same amount of cobalt of (**a**) 6 mC, (**b**) 12 mC and (**c**) 24 mC are compared with the curve of the GC/Ag12 sample of [31]. All curves are recorded at 500 rpm with a scan rate of 50 mV·s^−1^.

Figure 7 shows that the addition of Co enhances the catalytic effect on ORR, even with respect to Ag(111), and that the maximum effect is shown by the addition of 12 mC of Co. A still more interesting comparison is that offered by Figure 8a–c, where the ORR curves obtained on Ag are reported together with those recorded after the addition of 6, 12 or 24 mC of Co and together with the same amounts of Co, but without Ag. These figures show that in all cases, the ORR recorded on Co alone starts before that recorded on Ag, probably because at the very beginning of the reduction curve, the autocatalytic mechanism prevails. However, going on towards the negative values, the current involved is lower than on Ag alone, because of the higher catalytic effect of silver. In all cases, the contemporary presence of Co and Ag gets the best of both, so that not only the ORR is anticipated, but also the limiting cathodic current increases, thus indicating a higher number of electrons involved.

**Figure 9:**
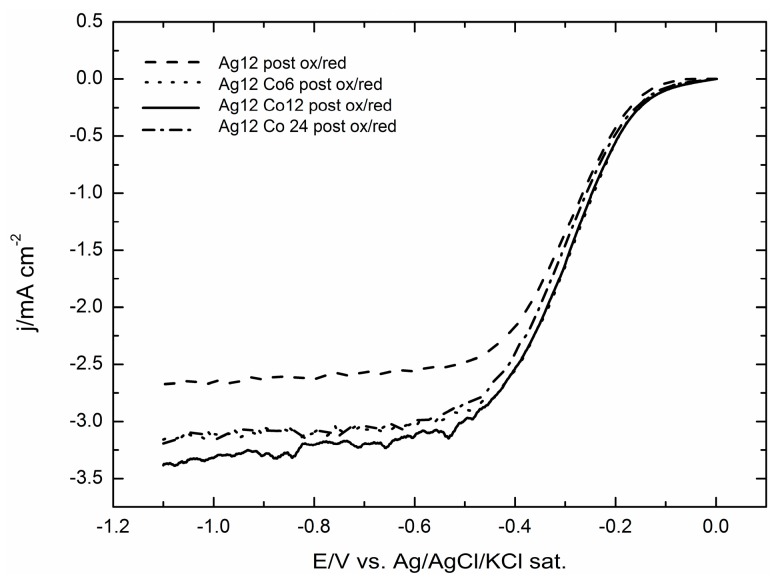
The effect of the oxidative/reductive treatment on the ORR of the GC/Ag of [31] and GC/AgCo samples recorded at 500 rpm and with a scan rate of 50 mV·s^−1^.

Actually, as reported in [31], the catalytic effect of the silver microparticles deposited on GC substantially improves with an activation protocol based on pretreatment oxidation/reduction cycles. We tentatively attributed the enhancement of the catalytic effect to the formation of Ag–OH, which is assumed to be active towards ORR catalysis [21]. Therefore, we applied the same activation protocol to the deposits containing both silver and cobalt, and of course, we compared the ORR curves with that of the activated microparticles of silver alone reported in [31]. Figure 9 shows that the addition of Co still enhances the catalytic effect and that the best curve is still that corresponding to the addition of 12 mC of Co to the deposit of 12 mC of Ag.

**Figure 10:**
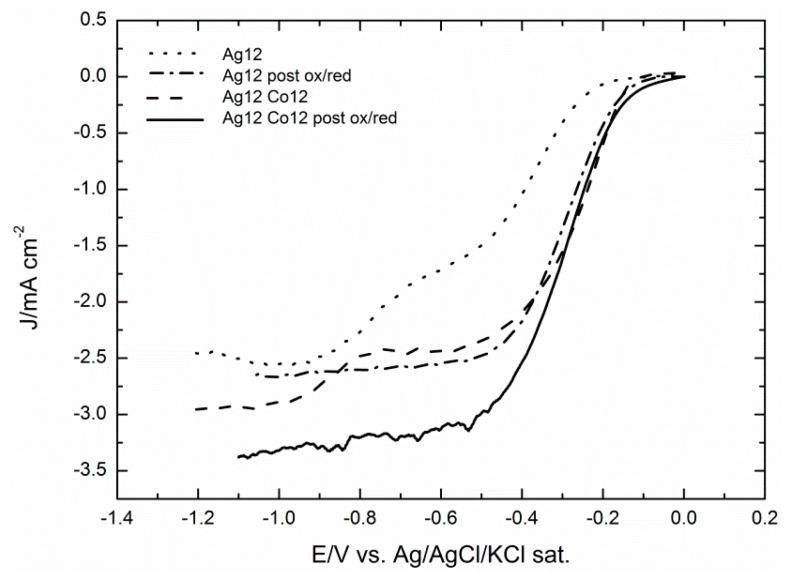
ORR of the best performing catalyst made by GC/Ag12Co12 before and after the oxidative/reductive treatment recorded at 500 rpm and with a scan rate of 50 mV·s^−1^. The curves are compared to the GC/Ag samples of [31].

For a more detailed analysis, Figure 10 stresses the strong enhancement imparted by the pretreatment protocol, both on the deposits of silver alone and on the deposits also containing cobalt.

**Figure 11:**
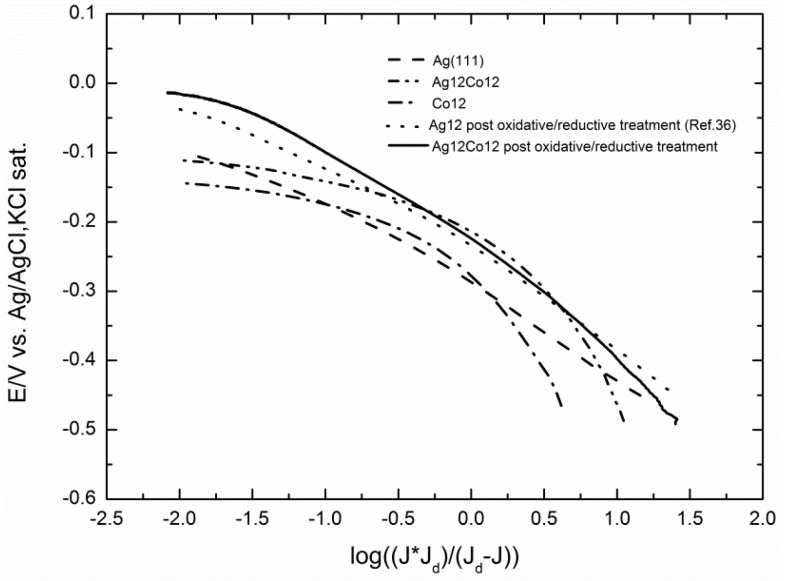
The Tafel plots for the ORR of the GC/Co, GC/Ag12Co12 before and after the oxidative/reductive treatment are compared to the Tafel plot of GC/Ag12 sample reported in [31] and the Tafel plot of a Ag(111) electrode.

Tafel plots reported in Figure 11 show that the shape of the activated curve of the Ag/Co deposit (solid curve) is similar to that of the activated Ag (dotted curve), thus denoting that the same reaction mechanism is operating.

Both curves are linear over large potential intervals and exhibit slopes very close to that of Ag(111) (dashed curve) [8,31]. It is worthwhile to note that the Tafel plot of the not pre-treated Ag/Co sample appears to be a continuous curve for which it is impossible to determine a single slope value. This shape is similar to that of a deposit of Co alone, with the only advantage of a positive shift of the reduction potential already observed in Figure 8a–c. As already reported in [28], such a different shape of the Tafel plots should rule out that the enhanced catalytic effect could be solely ascribed to a mere increase of the electrode area as a consequence of the surface roughening during the pretreatment and that it should be better ascribed to the formation of oxydril-containing species.

The number of electrons transferred per O_2_ molecule can be estimated using either the Levich or the Koutecky–Levich equation on ORR curves obtained at different rotation rates. More precisely, the Levich plot is used when the process is diffusion controlled by the very beginning of the reduction curve, whereas the Koutecky–Levich plot better accounts for the kinetic contributions that are responsible for the deviation of the curve from the characteristic sigmoidal shape.

**Figure 12:**
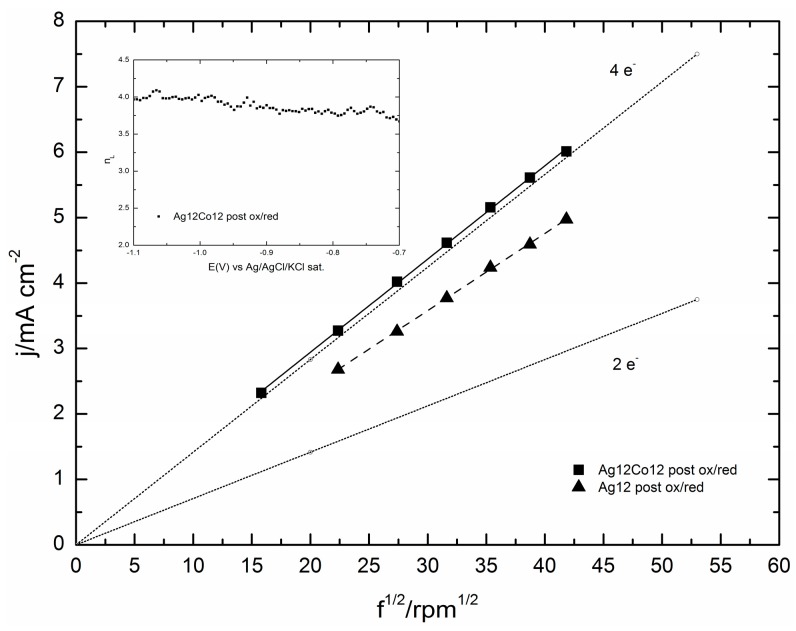
The Levich plot of the GC/Ag12Co12 after the oxidative/reductive treatment and the GC/Ag12 sample after the same treatment at E = −1.1V is reported. Inset: the average number of electrons exchanged during the ORR and extrapolated from the Levich plot of the GC/Ag12Co12 sample is reported in the potential range −0.8–−1.1 V.

As shown by Figure 9, the shape of ORR curves registered on the sample containing either Ag or Ag/Co, both of them undergoing the oxidation/reduction pretreatment, justifies the use of the Levich equation:

(6) $$j_{d}=\text{0.620 n F }{\text{D}}_{\text{O}}^{\text{2/3}}\nu^{-1/6}C_{O}\omega^{1/2}$$

where j_d_ is the diffusion limited current density, n is the number of exchanged electrons, F is the Faraday constant, D_0_ is the oxygen diffusion coefficient (1.95 × 10^–5^ cm^2^/s) [35], ω is the angular rotation rate of the electrode (in rad/s), ν is the kinematic viscosity of the solution (0.008977 cm^2^/s) and C_0_ is the O_2_ solubility in solution (1.15 × 10^−3^ mol/dm^3^) [35]. Figure 12 shows that in the potential region between −0.8 and −1.1 V, the plots of j_d_ against f^1/2^, where f is the angular rotation rate of the electrode in rpm, are straight lines intersecting the origin and with slopes yielding n values ranging between 3.22 and 3.53 for the Ag sample and 3.8 and 4.03 for Ag/Co (see the inset of Figure 12). This result justifies the increase in the cathodic current density of the Ag/Co sample observed in Figure 9 well.

### 2.3. Electrocatalytic Evaluation

The catalytic activity of a catalyst may be verified by parameters, such as the electrochemically-active surface area (ECSA), specific activity and mass activity [40,41]. In our case, the analysis was carried out on the silver microparticles of Figure 9 at the potential E_1/2_ = −0.29 V (equal to 0.68 V versus the reference hydrogen electrode (RHE)), re-examining the experimental data of [31]. There, the real area had been determined by depositing Pb at underpotential and measuring the charge involved in its re-dissolution. Thus, a real area of 1.75 cm^2^ was found for a deposit of 12 mC of silver on a 0.785-cm^2^ GC electrode, corresponding to a silver loading of 13.4 μg. Once corrected for the roughness factor, the silver loading yields an ECSA value equal to 13 m^2^·g^−1^.

Then, the kinetic current measured at −0.29V, I = 1 mA, was divided either by the silver loading or by the real area to get the mass activity, MA, or the specific activity, SA.

As shown in Table 2, our values for ECSA, SA and MA are of the same magnitude order of those reported in [41] for Pt/Vulcan catalysts. Of course, as expected, the whole ORR curve on silver is shifted towards more negative potentials.

**Table 2:** Kinetic parameters of a commercial Pt/Vulcan catalyst and the Ag 12 post ox/red sample: electrochemical surface area (ECSA) half wave potential (E_1/2_), specific activity (SA_E1/2_) and mass activity (MA_E1/2_).

Samples	ECSA (m^2^·g^−1^)	E_1/2_ (mV) *vs.* RHE	SA_E1/2_ (mA·cm^−2^)	MA_E1/2_ (A·g^−1^)
**Pt/Vulcan [42]**	44	854	0.21	92
**Ag 12 post ox/red**	13	680	0.57	75

The addition of cobalt strongly enhances the limiting current due to the significant increase in the number of electron, thus supporting the hypothesis of a synergic effect on silver.

## 3. Experimental Section

Merck analytical reagent-grade AgNO_3_, CoSO_4_, Na_2_S_2_O_3_ and KOH were used without further purification. Merk Suprapur HClO_4_ and Aldrich NH_4_OH were used to prepare the pH 9.2 ammonia buffer used as the supporting electrolyte. The solutions were freshly prepared just before the beginning of each series of measurements. An automated deposition apparatus consisting of Pyrex solutions reservoirs, solenoid valves, a distribution valve and a flow-cell was used under the control of a computer. The working electrode was SIGRADUR^®^ G Glassy Carbon from the HTW Hochtemperatur-Werkstoffe GmbH Company (Thierhaupten, Germany). The electrolytic cell used for the electrodeposition study was a Kel-F cylinder with a 10-mm inner diameter, delimited by the working electrode (a disk of 15 mm in diameter) on one side and the counter electrode on the other side. The resulting geometric area of the electrode is 0.785 cm^2^. The inlet and the outlet for the solutions were placed on the sidewalls of the cylinder. The counter electrode was a gold foil, and the reference electrode was an Ag/AgCl/KCl saturated electrode placed on the outlet tubing. Both the distribution valve and the cell had been designed and realized in the workshop of our department [42]. The solution is pushed into the cell by applying a pressure as low as 0.3 atm that determines a flow-rate of about 1 mL·s^−1^. After filling the cell, the pressure is no longer applied, so that the flow stopped during deposition. Simple homemade software allows for filling the cell with the various solutions and controls the deposition by keeping the potential at the planned value for the time necessary to deposit the programmed amount of silver.

Before silver and cobalt depositions, the working electrode was mechanically polished with alumina powders of decreasing size down to 1 μm. After each step of the abrasion procedure, the electrode was washed with bidistilled water. If already used in preceding measurements, the electrode was treated with an additional step of chemical etching based on a mixture of H_2_O_2_ and NH_3_ that ensured the dissolution of any remaining traces of silver. An electrochemical activation of the GC surface was made inside the electrolytic cell by alternating the application of potentials as negative as −1.6 V and as positive as +1.5 V, keeping the electrode at the selected potential for 60 s while rinsing the cell with ammonia buffer every 30 s. The electrode surface final check, carried out recording a cyclic voltammogram in ammonia buffer, showed a flat double layer region between +0.8 and −1.2 V, approximately.

After each silver and/or cobalt deposition, the electrode was transferred to the electrolytic cell used for the Rotating Disk Electrode (RDE) catalytic measurement that was a Metrohm glass cell equipped with 4 glass ports (Herisau, Switzerland). The working GC-modified electrode placed on a Teflon support realized in the workshop of our department was connected to an AutoLAB RDE-2 support rotating electrode managed by an AutoLAB manual motor controller. An Ag/AgCl/KCl saturated) reference electrode and a Pt wire counter electrode were used. A glass bubbler connected to an O_2_ bottle was used to saturate the KOH 0.1 M electrolyte solution.

## 4. Conclusions

The behaviour of cobalt deposition was investigated both on GC and on microparticles of silver obtained depositing 12 mC of silver on a 0.785-cm^2^ GC electrode. Aiming at depositing very small amounts of either silver and/or cobalt, we used a purpose-developed program that allowed for strictly controlling the amount of deposition, resulting in a good reproducibility of all reported experiments.

The polarization curves recorded on a rotating-disc working electrode for the ORR on increasing deposits of cobalt on GC show that the onset potential progressively shifts towards less negative potentials up to a deposit equal to 18 mC (equal to 7.0 μg cm^−2^), and then, it progressively shifts back to the negative potentials for the largest Co deposits. At the same time, the limited cathodic currents follow a similar behaviour. This is well depicted by both the plots of the potentials at which the curves reach the value of 0.5 mA cm^−2^ and the plots of the currents recorded at a potential E = −1.0 V. The results are volcano curves that are typical of a mechanism where the adsorbed step is the rate-determining one. At the same time, the cyclic voltammograms recorded at different scan rates, v, at the GC/Co(OH)_2_ electrode allow one to verify that i_p_/v decreases with v, thus confirming the autocatalytic mechanism imparted by the presence of cobalt as reported in [35]. Tafel plots of all Co deposits are continuous curves for which it is impossible to determine a single slope value (see Figure 11 for the Co deposit of 12 mC).

Amounts of Co corresponding to 6, 12 and 24 mC were added to the silver deposit of 12 mC (corresponding to 17 μg·cm^−2^) that had given the best catalytic effect in [31]. In all cases, the addition of Co enhances the catalytic effect of silver alone on ORR even with respect to Ag(111) (Figure 7). The comparison with the corresponding ORR curves of Co alone shows that the various ORRs of Ag/Co not only seem to combine the behaviours of Ag and Co alone, but also result in final synergic enhancements. Thus, all Ag/Co ORRs are anticipated, probably because at the very beginning the contribution of the autocatalytic mechanism of Co prevails, but going on towards the negative values, the curves feel the higher catalytic effect of silver and, finally, reach a higher limiting cathodic current, thus indicating a higher number of electrons involved. However, Figure 11 shows that the Tafel plot of Ag/Co maintains the curved shape of Co alone.

A further enhancement of the catalytic effect is obtained when the Ag/Co deposits undergo the activation protocol based on pretreatment oxidation/reduction cycles, as those described in [31] for the microparticles of silver alone. Together with the immediate observation of more regular shapes of the ORR curves with higher limiting cathodic currents (Figure 9), the Tafel plots of the pre-treated Ag/Co deposit are linear over large potential intervals and exhibit slopes very close to that of the pre-treated Ag alone and of Ag(111). This circumstance indicates that the same reaction mechanism is operating, even though the higher limiting cathodic currents suggest a higher number of electrons involved, as confirmed by the Levich analysis.

The electrochemical investigation is able to give detailed indication of the catalytic effect. However, we hope that the morphological and structural characterization that is now in progress will be able to explain how the synergy works [43].
